# *Anopheles *larval abundance and diversity in three rice agro-village complexes Mwea irrigation scheme, central Kenya

**DOI:** 10.1186/1475-2875-9-228

**Published:** 2010-08-09

**Authors:** Joseph M Mwangangi, Josephat Shililu, Ephantus J Muturi, Simon Muriu, Benjamin Jacob, Ephantus W Kabiru, Charles M Mbogo, John Githure, Robert J Novak

**Affiliations:** 1 Kenya Medical Research Institute, Centre for Geographic Medicine Research - Coast, P.O. Box 428, Kilifi 80108, Kenya; 2 International Centre for Insect Physiology and Ecology, Nairobi Kenya; 3 Jomo Kenyatta University of Agriculture and Technology, Department of Zoology, Nairobi, Kenya; 4 Illinois Natural History Survey, University of Illinois, 1816 South Oak Street, Champaign, IL 61820, MC-652, USA; 5 Department of Medicine, William C. Gorgas Center for Geographic Medicine, 206C Bevill Biomedical Research Building, 845 19th Street South, Alabama, USA; 6 Kenyatta University, Department of Pathology, Nairobi, Nairobi, Kenya

## Abstract

**Background:**

The diversity and abundance of *Anopheles *larvae has significant influence on the resulting adult mosquito population and hence the dynamics of malaria transmission. Studies were conducted to examine larval habitat dynamics and ecological factors affecting survivorship of aquatic stages of malaria vectors in three agro-ecological settings in Mwea, Kenya.

**Methods:**

Three villages were selected based on rice husbandry and water management practices. Aquatic habitats in the 3 villages representing planned rice cultivation (Mbui Njeru), unplanned rice cultivation (Kiamachiri) and non-irrigated (Murinduko) agro-ecosystems were sampled every 2 weeks to generate stage-specific estimates of mosquito larval densities, relative abundance and diversity. Records of distance to the nearest homestead, vegetation coverage, surface debris, turbidity, habitat stability, habitat type, rice growth stage, number of rice tillers and percent *Azolla *cover were taken for each habitat.

**Results:**

Captures of early, late instars and pupae accounted for 78.2%, 10.9% and 10.8% of the total *Anopheles *immatures sampled (n = 29,252), respectively. There were significant differences in larval abundance between 3 agro-ecosystems. The village with 'planned' rice cultivation had relatively lower *Anopheles *larval densities compared to the villages where 'unplanned' or non-irrigated. Similarly, species composition and richness was higher in the two villages with either 'unplanned' or limited rice cultivation, an indication of the importance of land use patterns on diversity of larval habitat types. Rice fields and associated canals were the most productive habitat types while water pools and puddles were important for short periods during the rainy season. Multiple logistic regression analysis showed that presence of other invertebrates, percentage *Azolla *cover, distance to nearest homestead, depth and water turbidity were the best predictors for *Anopheles *mosquito larval abundance.

**Conclusion:**

These results suggest that agricultural practices have significant influence on mosquito species diversity and abundance and that certain habitat characteristics favor production of malaria vectors. These factors should be considered when implementing larval control strategies which should be targeted based on habitat productivity and water management.

## Background

Irrigation development projects have been associated with negative impacts on human health, particularly with respect to vector-borne diseases. There is evidence for direct relationship between irrigation development and increased malaria transmission [1,2]. Rice fields have proved to be particularly well suited as larval sites for *Anopheles gambiae s.l.*, the main malaria vector in sub-Saharan Africa. This heliophilic species thrives in the shallow inundated fields during tilling, transplanting, the first 6 weeks of the growing period (until canopy closure), and after harvest [3]. Therefore, in addition to nutritional and socio-economic benefits associated with irrigated rice cultivation [4-6], this also comes along with the creation of large and more permanent larval habitats that support higher densities of malaria vectors. Such observations have been made in Kenya [7-12], Burkina Faso [13], The Gambia [14], Madagascar [15], Senegal [16] and Mali [4]. However, the impact of irrigated rice cultivation on malaria transmission is controversial [5] with some cases reporting increasing in mosquito densities while others reporting reduction. However, studies across Africa have demonstrated the negative impact of irrigation on mosquito borne diseases. Although introduction of irrigated agriculture has little or no impact on malaria transmission in areas of stable transmission,[5,17] irrigated rice cultivation in semi-arid savannah zone of Africa can alter malaria transmission pattern from seasonal to perennial [18,19]. An increase in the density of *An. gambiae s.l*., *Anopheles funestus*, and *Culex quinquefasciatus *and a consequent increase in the prevalence of Bancroftian filariasis has also been reported after introduction of irrigated agriculture [20-24]. Thus proper understanding of the factors that promote mosquito production may provide useful information on how to mitigate the negative effects of irrigated rice cultivation on human health.

Variation in distribution and abundance of *Anopheles *population observed in the above studies may reflect the oviposition preferences of gravid mosquitoes and the ability of immature stages to tolerate the conditions that prevail within their aquatic habitats. In-depth understanding of ecological characteristics of the larval habitats and the factors affecting vector abundance is fundamental in developing vector control methods particularly in rice agro-ecosystems where inundated rice fields are associated with higher vector densities. Physical factors such as habitat stability or degree of spatial heterogeneity and biotic factors such as predation are also known to influence mosquito species assemblages [25,26]. Effective control of malaria through vector management requires information on the distribution and abundance of aquatic habitats and relative densities of vectors in targeted areas.

Larval control is a potentially important target in malaria vector control. Source reduction through modification of larval habitats was the key to malaria eradication efforts in the United States, Italy, and Israel [27]. The suppression and even eradication of malaria from vast areas has been attributed to effective large-scale programs to kill immature *Anopheles *species vectors or reduce the number of suitable larval habitats around human dwellings [28]. The appropriate management of larval habitats particularly during the dry season may help suppress vector densities and consequently, malaria transmission [29-31]. Recent studies in sub-Saharan Africa (SSA) have shown that larval control using microbial larvicide is effective in reducing adult densities in several countries including Kenya [32-34], Eritrea[35], Tanzania [36], Gambia [37] and can work in synergy with insecticide-treated bed nets to significantly lower malaria transmission [38]. However, our understanding of anopheline larval ecology in rice agro-ecosystems is still insufficient and this affects the design and implementation of larval control. The objective of this study was to describe key anopheline larval habitats and determine the spatial and temporal heterogeneities in larval abundance in three villages with different agricultural practices. The goal of the study was to provide data that will make it possible to develop targeted and sustainable larval management strategies in irrigated rice agro-ecosystems in Africa.

## Methods

### Study area

The study was done in Mwea Irrigation Scheme (MIS), in Kirinyaga District approximately 100 km North East of Nairobi Kenya. This area has been previously described [9-11,39,40]. Mwea occupies the lower altitude zone of Kirinyaga District in an expansive low-lying area mainly characterized by black cotton soil. The mean annual rainfall for year 2004 was ≈ 850 mm with maximum amount falling in April to May (long rains) and October to November (short rains). The average temperatures are in the range of 16 - 26.5°C. Relative humidity varies from 52-67%. The study was conducted from April 2004 to March 2005. *Anopheles arabiensis *is the predominant vector of malaria, and the only sibling species of the *An. gambiae *species complex recorded in the area [9].

The study was conducted in 3 villages with distinct land use patterns: 1) Mbui Njeru village (1,100 m above sea level) is situated within the MIS (planned rice cultivation) and more than 80% of the area in this village is under rice cultivation. Farmers in this village adhere to the MIS irrigation calendar for water management and rice growing. The main rice growing season begins in June and ends in December while the second crop (ratoon) is grown between January and April. 2) Kiamachiri village (1,200 m above sea level) is located outside the MIS tenant farms and rice farming is approximately 25% of the total area. Individual farmers plan their own rice cropping cycle (unplanned rice cultivation) depending on availability of water either from rain or from small streams around the villages. Thus depending on availability of water, rice may be grown all year round. 3) Murinduko village (1,350 m above sea level), is a non-irrigated area outside the MIS. It is situated on the slopes of Murinduko hills and is served by two streams that flow at the edges of the village. The soil in this area is highly porous and seepage of rain water is quite high. Although it is a non-irrigated village, rice is grown on a small scale along the two streams. Only less than 5% of the total area is under rice cultivation.

The typical rice cultivation cycle includes land preparation (cultivation/rotavation), sowing - transplanting phase, a vegetative phase (including early and vegetative stages), flowering phase and maturation phase followed by harvesting.

### Rainfall and relative humidity

In each village, a rain gauge (Tru-Chek^®^, Rain Gauge Division, Edwards Manufacturing Co. Albert Lea, Minnesota, USA) was placed and read daily at 0900 hrs. A HOBO^® ^Micro Station (Onset Computer Corporation, Bourne, Massachusetts, USA) was setup at Mwea Irrigation and Agricultural Development (MIAD) Centre to further monitor rainfall, relative humidity and temperature. BoxCar Pro (Onset Computer Corporation, Bourne, Massachusetts, USA) was used to download the weather information every month end.

### Larval sampling and processing

Larval sampling processing was conducted as previously described [10]. All non-paddy larval habitats present within each village were identified, categorized, and sampled every two weeks for mosquito larvae over a 12-month period (April 2004-March 2005). Three, four, and five randomly selected paddy blocks 60 m × 60 m were also included and formed the sampling unit for the paddy habitat category in Mbui Njeru, Murinduko and Kiamachiri, respectively. The differences in the number of paddy blocks selected in each village were due to differences in the rice cropping cycles. Diverse larval habitat types present in each paddy block were identified and sampled separately for mosquito larvae. Up to 20 dips were taken at intervals along the edge of each larval habitat using a standard mosquito dipper (350 mL Bio Quip Products, Inc. California, USA) depending on the size of the aquatic habitat. For vegetated habitats, the vegetation was carefully opened up to allow for water pooling before dipping was done. The larvae for each habitat were placed separately in whirl paks and transported to the laboratory where they were sorted by genus and instar, counted, and recorded. All third and fourth instar larvae of the genus *Anopheles *were preserved in 100% ethanol and later identified morphologically to species [41]. Physical and biological characteristics of the larval habitats including habitat type, distance to the nearest house, surface debris, emergent, floating and submerged vegetation cover, turbidity, water depth, presence of *Azolla*, habitat stability (permanent or temporary), and presence or absence of other invertebrates were recorded. Water depth was measured using a metal ruler. Distance to the nearest homestead was measured using a tape measure if less than 100 m and estimated if more than 100 m. Distance was then categorized into 6 classes: 1) ≤100 m, 2) 101 to 200 m, 3) 201 to 300 m, 4) 301 to 400 m, 5) 401 to 500, 6) ≥500 m. Surface debris, emergent, floating and submerged vegetation cover, and presence of *Azolla *were estimated as percent of total surface covered [42,43]. Habitat stability was expressed in terms of the length of time the habitat contained water. A habitat was considered temporary if it held water for 2 weeks or less and permanent if it held water for more than 2 weeks. Turbidity was categorized into 4 classes namely clear, low, medium and high based on watercolor on a white background.

### Statistical analysis

Statistical analyses were done using SPSS software (Version 15.0 for windows, SPSS Inc., Chicago, IL). One-way Analysis of Variance (ANOVA) was used to compare the differences in larval abundance between sites and habitat-type. Where significant differences were observed, the means were separated by Tukey test. Pearson correlation was used to determine the association between *Anopheles *larval density and rainfall. The relative abundance of *Anopheles *was calculated as the number of larvae divided by the number of dips taken from each larval habitat, and then expressed as density per 10 dips. The dependent variable (relative abundance of *Anopheles *larvae) was log-transformed log_10 _(x + 1) to stabilize the variance and improve normality of distribution. Multiple step-up logistic regression analysis was used to determine the correlation between environmental and agricultural variables and the presence of *Anopheles *larvae in the rice field. The rice fields had water most of sampling occasions hence were used for the regression analysis. For each environmental and agricultural variable, simple correlation between larval abundance and individual parameters were first checked and only significant associations were further examined by step-up multiple logistic regressions to determine the best predictor variables associated with relative abundance of the larval species of anophelines. The results for the regression analysis were reported as significant if P < 0.05.

## Results

### Larval abundance

Captures of 1^st^-2^nd ^(early instars), 3^rd^-4^th ^(late instars) and pupae accounted for 78.2% (n = 22,885), 10.9% (n = 3,192) and 10.8% (n = 3,175), respectively of the total *Anopheles *immatures sampled (n = 29,252). There was a significant site-to-site variation in larval abundance and the Tukeys Honest Significant Difference (HSD) (α = 0.05) further indicates that the 3 villages are different from each other in larval abundance. Murinduko had significantly higher pupal counts compared to Mbui Njeru and Kiamachiri (ANOVA, F = 2.726, df = 2, p < 0.01).

In all the 3 villages, rice fields and canals had higher densities of anopheline larvae than the other habitat types (ANOVA, df 2, p = 0.000) (Table 1). Rice fields and canal were more productive in Murinduko compared to Mbui Njeru and Kiamachiri (F = 6.529, df = 11, *P *< 0.01). Peridomestic habitats (pools) in the 3 villages had higher densities of *Anopheles *larvae. Further analysis of the rice field habitats showed that early rice growth stages (land preparation, transplanting and tillering) had significantly higher *Anopheles *larval counts than the later stages (booting, flowering, maturation) (ANOVA df = 5, p < 0.001). Marshes were also important habitat type in the 3 villages in which they had high densities in Murinduko. Water reservoirs had high larval densities in Murinduko but in Mbui Njeru and Kiamachiri the reservoirs had lower densities. Murinduko had some special habitat categories such as tree holes and rock pools, which had anopheline larvae especially during the rain season. Although the tree holes and rock pools have few numbers of larvae, they had high pupal densities.

**Table 1:** Relative abundance of *Anopheles *larvae in different habitats found in the three villages

Village	Habitat type	Early stage *Anopheles*	Late stage *Anopheles*	Pupal stage
Mbui Njeru	Rice field	1,626	168	228
	Canal	372	32	8
	Pools	45	2	6
	Marsh	807	141	93
	Water reservoirs	8	0	137

Kiamachiri	Rice field	3,945	305	168
	Canal	856	59	83
	Pools	537	52	104
	Marsh	1,270	163	160
	Water reservoirs	17	2	21

Murinduko	Rice field	8,523	1,508	844
	Canal	718	188	93
	Pools	1,197	124	104
	Marsh	2,765	436	564
	Water reservoirs	121	2	457
	Tree holes	14	0	15
	Rock pool	64	10	88

### *Anopheles *species composition and distribution among the habitats

Morphological identification of late instars of *Anopheles *larvae (n = 1,893) yielded seven anopheline species comprising of *An. arabiensis *(82.1%), *An. pharoensis *(7.8%)*, An. funestus *(2.5%)*, Anopheles rivorulum *(2.1%)*, Anopheles maculipalpis *(2.0%)*, Anopheles rufipes *(2.6%), and *Anopheles coustani *(1.0%) (Table 2). All the 7 anopheline species were present in Murinduko and Kiamachiri, whereas only 3 species were represented in Mbui Njeru. The relative importance of aquatic habitats in supporting larval development of different anopheline species was variable amongst the villages. In Murinduko, *Anopheles *larvae were found in all the 5 habitat types sampled at this village. In the planned rice-growing village (Mbui Njeru), rice fields, canals and temporary pools produced only 3 anopheline species (*An. gambiae s.l., An. pharoensis*, *An. rivulorum*). In Kiamachiri, larvae were found in 4 habitat types including rice fields, canal, marsh and temporary pools.

**Table 2:** Species distribution of 3^rd ^and 4^th ^stage *Anopheles *larvae in different larval habitats in 3 villages in Mwea (April 2004 - March 2005)

Village	Habitat type	*An. arabiensis*	*An. funestus*	*An. pharoensis*	*An. rivulorum*	*An. Maculipalpis*	*An. rufipes*	*An. coustani*	Total identified
Kiamachiri	Rice field	228	5	13	5	1	0	0	252
	Canal	25	0	1	1	0	0	1	28
	Marsh	26	1	1	0	0	0	0	28
	Temporary pool	107	4	8	1	1	4	1	126

Mbui Njeru	Rice field	84	0	4	1	0	0	0	89
	Canal	24	0	6	0	0	0	0	30
	Temporary pool	66	0	4	0	0	0	0	70

Murinduko	Rice field	871	33	92	18	38	19	9	1,080
	Canal	165	2	6	2	1	0	4	180
	Marsh	46	3	4	5	0	14	1	73
	Stream pool^a^	92	6	19	14	2	15	0	148
	Temporary pool	166	4	22	0	3	7	7	209
	Rock pool	0	0	0	0	0	0	0	0

Total		1,900	58	180	47	46	59	23	2,313

^a ^Stream pool denotes puddles on stream edges and on stream beds

### Habitat diversity

The different larval habitats encountered in the study sites included water canals, marshes, rice fields, temporary pools, water reservoirs, rock pools, stream pools, tree holes. Only 5 larval habitat types were identified in Mbui Njeru and Kiamachiri. In Murinduko, larval development was supported in 8 habitat types. A total of 226 larval habitats were sampled in Kiamachiri, 201 in Mbui Njeru, and 170 in Murinduko during the sampling period. The period of active productivity of the larval habitats, based on the proportion of sampling efforts when the habitats had water and proportion of times the habitat was positive for anopheline larvae was variable between sites (Table 3). Among the stable aquatic habitat categories (rice fields, canals and marshes), rice fields and associated canals had high densities of anopheline larvae in three villages, whereas the marshes had the highest *Anopheles *density in Murinduko (Table 3).

**Table 3:** Habitat dynamics, productivity and diversity of *Anopheles *larval habitats sampled over 12 months in three ecologically varied villages in the Mwea Rice Irrigation Scheme (April 2004 - March 2005)

Village	Habitat type	# Habitats sampled	% Larval habitats had water	# Sampled	% Larval habitats positive for *Anopheles *larvae	% *Anopheles *larvae alone	Larval density (no./10 dips)	Pupal density (no./10 dips)
Mbui Njeru ("Planned" rice cultivation; >80% area under rice)	Canal	19	83.0	225	31.6	13.7	1.1	0.0
	Marsh	6	19.5	17	47.1	21.2	1.7	0.2
	Rice field	80	57.6	545	46.8	28.4	2.2	0.4
	Temporary pool	96	14.4	374	36.1	30.5	2.4	0.2
	Water reservoir	2	23.1	6	16.7	5.7	0.4	5.6

Kiamachiri ("Unplanned" rice cultivation; ≈ 25% area under rice)	Canal	30	62.7	330	39.7	16.6	1.9	0.2
	Marsh^a^	27	48.3	193	48.7	18.3	2.1	0.3
	Rice field	108	52.7	749	56.3	35.2	4.1	0.2
	Temporary pool^b^	59	50.0	423	44.9	23.8	2.8	0.4
	Water reservoir^c^	2	46.2	24	25.0	5.8	0.7	0.7

Murinduko (Subsistence farming; < 5% area under rice on valley bottoms)	Canal	9	97.1	133	65.4	12.4	6.7	0.7
	Marsh	22	85.7	162	79.6	14.5	7.8	2.7
	Rice field	83	86.4	1126	69.3	16.3	8.8	0.7
	Rock pool	4	35.5	11	72.7	10.9	5.9	15.9
	Stream pool	10	93.9	108	76.9	15.8	8.5	0.6
	Temporary pools	35	90.7	234	76.9	18.3	9.9	0.6
	Tree hole	3	44.4	4	75.0	5.3	2.9	3.8
	Water reservoir	4	61.1	21	28.6	6.3	3.4	21.6

^a ^Marshes included seeps and swamps; ^b ^Temporary pools were represented by hoof prints, ditches, puddles, pits, tire tracks and quarries; ^c ^Water reservoirs represented tanks and wells

In Kiamachiri (unplanned rice cropping), rice fields were the most predominant habitat with a density of 4.1 larvae/10 dips followed by temporary pools (2.8 larvae/10 dips) and marshes (2.1 larvae/10 dips). The paddy contributed 35.2% of total *Anopheles *larvae collected in this site and the habitat type had water 56.3% of the total times assessed. In Mbui Njeru, other than the temporary pools (2.4 larvae/10 dips), rice fields (2.2 larvae/10 dips) were equally productive with the habitat having water 57.6% of the times monitored.

The temporary aquatic habitats (temporary pools, water reservoirs/tanks, tree holes and rock pools) showed variable significance but had the highest pupal and larval density during the period assessed. The importance of these temporary larval habitats was limited since the habitats were dry 85.7% of the times monitored. Although canals in the 2 rice growing sites had generally low larval densities, they were active almost over the 12 months of the study as they held water 83.0% and 67.2% of the times surveyed in Mbui Njeru and Kiamachiri, respectively, and were positive for anopheline larvae approximately 40% of the sampling effort. Water reservoirs including drainage channels at water collection points were only of limited significance in the 2 sites. Murinduko, with less than 5% of area under rice, had the highest representation of larval habitat types with the highest larval density obtained from the temporary pools (9.9 larvae/10 dips). Larval production from canals, marshes, rice fields and stream pools was not highly variable (range: 6.7 - 8.8 larvae/10 dips). Rock pools (n = 4) and tree holes (n = 3), for *Anopheles *breeding, were only encountered in Murinduko.

### Temporal variation in habitat preference and larval density

Larval density was highly variable in different habitat types and during the seasons. The peak of larval production was associated with rice cropping patterns and rainfall. The rice-cropping pattern was based on the calendar of Mwea Rice Irrigation Scheme. The peak larval production in rice fields was recorded from September to October in Mbui Njeru, and September to March in Kiamachiri (Table 4). In Murinduko larval densities within the rice fields was high for longer periods of time (12 months) with a peak in September. The marshes and canals had higher larval densities in the 3 study villages although the period of active production of larvae was variable. While marshes had high larval densities almost throughout the year in Murinduko (May - March) they had high larval densities in March only in Mbui Njeru and November to March in Kiamachiri. Temporary pools were generally mostly present between April - June and November - January in Mbui Njeru and Kiamachiri, respectively. In Murinduko, the stream pools were productive throughout the year. Rock pools, which were only encountered in Murinduko, were active (with water) over 2 periods (April - July; October - December) coinciding with the rains (Table 4). Water reservoirs were only with water for limited time over the 12 months study period.

**Table 4:** Seasonal variation in *Anopheles *larval densities over a 12-month sampling period among different aquatic habitats in three study sites in Mwea, Kenya (*Density is expressed as number of larvae per 10 dips*)

		Month												
			
Village	Habitat type	Apr	May	Jun	Jul	Aug	Sept	Oct	Nov	Dec	Jan	Feb	Mar	Mean
Mbui Njeru	Canal	0.7	0.7	0.1	1.1	0.3	2.5	1.3	1.6	1.4	1.1	1.0	1.0	1.05
	Marsh	0.0	0.0	0.2	^a^-	0.7	-	2.1	1.0	0.0	-	0.7	16.7	1.78
	Rice field	2.2	0.7	1.0	0.7	1.4	6.0	3.0	0.4	4.2	2.0	3.0	4.4	2.41
	Temporary pool	7.9	2.9	0.5	-	0.9	0.7	0.5	1.1	1.8	0.3	0.1	0.5	1.42
	Water reservoir	0.0	-	-	-	-	-	0.0	0.0	2.7	-	-	0.0	0.22
	Rainfall	205.6	47.3	8	0	0	18	141.8	231	63.5	22.5	16.9	48.5	66.93
	
	**Total larvae**	**10.8**	**4.3**	**1.8**	**1.8**	**3.3**	**9.2**	**6.9**	**4.1**	**10.1**	**3.4**	**4.8**	**22.6**	**6.88**

Kiamachiri	Canal	1.4	0.1	0.4	1.1	2.0	2.8	4.9	3.7	1.7	3.0	1.3	1.4	1.99
	Marsh	1.1	0.5	1.2	2.7	2.7	1.8	1.3	4.2	4.0	2.5	2.2	2.0	2.18
	Rice field	2.1	1.3	1.7	1.4	1.8	7.9	4.5	7.3	8.6	9.8	3.0	6.2	4.64
	Temporary pool	1.9	2.0	1.8	0.9	0.2	3.2	0.6	6.9	2.8	2.4	1.7	0.7	2.09
	Water reservoir	0.2	0.0	1.3	0.0	-	-	1.0	1.8	0.0	2.0	0.0	-	0.52
	Rainfall	181.8	74.8	28	8	1.6	71	96.2	136	47.5	21	1	13	56.66
	
	**Total larvae**	**6.7**	**3.9**	**6.4**	**6.1**	**6.7**	**15.7**	**12.3**	**23.9**	**17.1**	**19.7**	**8.2**	**10.3**	**11.42**

Murinduko	Canal	2.5	2.8	4.9	4.9	17.9	8.0	3.4	1.8	2.9	3.9	4.9	8.9	5.58
	Marsh	2.3	5.7	6.8	3.9	8.2	27.1	9.4	7.9	3.9	4.2	6.6	8.5	7.86
	Rice field	2.1	3.8	11.3	6.1	12.0	16.3	6.9	5.1	4.1	6.9	7.4	11.2	7.77
	Rock pool	3.0	3.4	-	-	-	-	13.0	9.5	3.5	-	-	-	2.70
	Stream pool	7.6	3.5	10.5	3.9	11.8	5.0	3.0	3.5	5.7	8.6	14.2	14.3	7.64
	Temporary pool	8.5	1.4	12.7	7.1	6.8	11.0	9.3	21.9	8.6	9.2	8.8	5.0	9.18
	Tree hole	0.0	0.0	0.0	-	-	-	0.0	0.0	0.8	9.0	-	-	0.82
	Water reservoir	0.5	0.0	-	-	-	-	0.0	8.7	9.0	0.0	0.0	0.0	1.51
	Rainfall	119	56	10	0	9	15	190	324	54	6	4	23	67.5
	
	**Total larvae**	**26.5**	**20.6**	**46.2**	**25.9**	**56.7**	**67.4**	**45.0**	**58.4**	**38.5**	**41.8**	**41.9**	**47.9**	**43.06**

^a^- (Minus sign) indicates that no aquatic habitat was present

Rainfall was highest in the three villages between March and June (Long rains) and October and December (Short rains). Results of Pearson correlation analysis showed that the *An. arabiensis *larval abundance was positively correlated with the both short and long rains in Kiamachiri (r = 0.759) and short rains in Mbui Njeru (r = 0.602). In contrast, larval abundance was negatively associated with both the long and short rains in Murinduko (r = -0.267).

### Factors associated with habitat preference

Multiple logistic regressions showed that turbidity, water depth, presence of other invertebrates, percentage *Azolla *cover, and distance to nearest homestead were the best predictors for *Anopheles *mosquito larval abundance in the habitats (Table 5). Turbidity and depth of the habitat had a positive association with the larval abundance while presence of other non-mosquito invertebrates, percent *Azolla *cover and distance to the nearest homestead or house had a negative effect on the larvae. Water turbidity was an important indicator of larval abundance, whereby habitats with clear or low turbidity tended to harbor most of the anopheline larvae. The percent *Azolla *cover had a negative effect on the *Anopheles *larvae abundance, when the *Azolla *cover increased in the habitats, low numbers of *Anopheles *larvae were present.

**Table 5:** Logistic regression for *Anopheles *larval abundance in the rice field habitat type in the 3 agro-village complexes in Mwea Kenya

Variable	B	**S.E**.	df	**Sig**.	OR (95% CI)
Turbidity			2	0.004	0.690 (0.537 - 0.888)
Clear	0.896	0.274	1	0.001	2.282 (1.345 - 3.872)
Low	0.731	0.246	1	0.003	1.705 (1.055 - 2.757)
Emergent vegetation	-0.156	0.232	1	0.499	0.938 (0.579- 1.519)
Floating vegetation	-0.294	0.205	1	0.152	0.727 (0.481-1.099)
Submerged vegetation	-0.088	0.878	1	0.921	0.745 (0.127-4.369)
Depth	0.048	0.020	1	0.016	1.047 (1.007-1.089)
Other invertebrates	-0.865	0.156	1	0.000	2.173 (1.596-2.959)
Rice height	-0.003	0.003	1	0.417	0.998 (0.991-1.005)
Tillers	-0.001	0.009	1	0.936	1.002 (0.983-1.022)
Water cover	-0.003	0.003	1	0.306	0.997 (0.992-1.002)
%Azolla	-0.010	0.002	1	0.000	0.990 (0.985-0.994)
Distance	0.012	0.003	2	0.000	1.012 (1.005-1.018)
0 - 100	-0.726	0.180	1	0.000	2.103 (1.196-2.551)
101 - 200	-0.599	0.176	1	0.001	1.537 (1.113 - 2.123)
Constant	1.010	0.399	1	0.011	

## Discussion

Understanding larval habitat ecology is important in designing targeted malaria control programs. This helps in knowing when a larval habitat is most productive and clearly shows when it should be targeted for maximum reduction in adult population. Currently there is renewed interest in mosquito larval control and the feasibility of reducing malaria vector populations through environmental and agro-ecosystem management approaches is currently being explored [9,44,45]. The ecologies of larval habitats were studied in 3 ecologically diverse villages based on rice cultivation patterns and water management in order to understand the variation in larval habitat dynamics and productivity. Both rice growing and rainfall significantly contributed to high abundance of mosquito larvae but the importance was highly site-specific. In, the 'planned' rice growing system (Mbui Njeru) larval abundance and densities corresponded well with the rice-growing season. Larval habitats in villages with 'unplanned' rice growing (out-growers) tended to have higher larval densities than the village with 'planned' (organized) rice growing an indication of diverse larval sites in these villages. The rice fields and canals in the 'unplanned' rice system were poorly drained making them more favorable for anopheline larval development, whereas the rice fields and the irrigation canals in the planned rice growing were well drained. The effect of unplanned rice growing and the subsequent uncoordinated water management meant that rice growing was undertaken throughout the year with rice fields at different rice growth stages throughout the year. This phenomenon not only increased the number of habitats but prolonged the period of productive life of the larval habitat for *Anopheles *larvae. Studies in rice growing irrigation schemes have shown that early stages of rice growth have been associated with high densities of mosquito larvae [3,11,39].

In Murinduko village rice cultivation has only recently been introduced along the river valleys. This has resulted in an increase in breeding sites for mosquitoes. Initially most of the habitats were concentrated on stream edges and stream pools. The soils and topography of this village does not allow formation of rain-fed pools hence the negative association between rainfall and larval densities. Species diversity was higher in this village than in the other villages suggesting the presence of diverse and productive larval habitats in this site compared to the rice growing villages where larval breeding is mainly limited to rice fields and associated canals [46]. For example, studies in western Kenya have shown similar trends where greater assemblages of anopheline species were associated with villages in rice growing which had permanent and diverse larval habitats [7,20,47,48].

*Azolla *cover was negatively associated with anopheline larval abundance. *Azolla *provides a mat-like structure on the surface of the habitat thus reducing penetration of sunlight which in turn affects photosynthetic activity of algae and other aquatic forms that serve as a food source for mosquito larvae [49,50]. The macrophyte mat may also inhibit oviposition in these habitats. The negative effect of the *Azolla spp *on mosquito production has been documented by other investigators [43,51]. Increase in turbidity resulted in a significant increase in anopheline larval densities in the habitats. It is likely that increase in turbidity tended to affect the attractiveness of these breeding sites to ovipositing female *Anopheles *mosquitoes. McCrae [52] showed that *An. gambiae *females preferred to oviposit on turbid water rather than on clear water. Several factors contribute to turbidity including insoluble particles of soil, organics, microorganisms, and other materials. The results of this study indicate that larval *An. arabiensis *are more abundant in relatively clear water than in turbid water which is unlike the studies by McCrae [52]. This results are similar to the findings of earlier studies in Kenya [42,53,54]. In rice fields, turbidity of water results from agronomic activities such as manual weeding. It was observed that top dressing with nitrogenous fertilizers lowered turbidity of water and it corresponded with increase of mosquito larvae [11,55-57]. Presumably gravid mosquitoes using visual cues viewed the dark substrate of mud soil through the clear water and this attracted them to oviposit in these habitats. Rice is grown in water maintained at a depth between 3 and 10 cm in Mwea Irrigation Scheme. The shallow waters would enable gravid mosquitoes to view the substrate, considering that *An. arabiensis *prefer shallow habitats [41,42,53].

Larval counts decreased with increasing distance from the homesteads. Studies have shown that *An*. *arabiensis *feeds predominantly on cattle and humans [9]. Gravid mosquitoes may utilize the habitats within close proximity to the homesteads for oviposition as an evolutionary strategy for energy conservation. Presence of other invertebrates was negatively associated with anopheline larval abundance. The invertebrate composition in the habitats may have been important in predation, which has a negative effect on the populations. The negative association between presence of other invertebrates and larval densities indicated the role natural regulation has in controlling vector production. Gravid mosquitoes might choose habitats with fewer densities of other non-mosquitoes invertebrates as a strategy to ensure that their progeny survives well with little risk of predation.

The data generated from this study suggest that implementation of larval control activities should be targeted based on habitat productivity, which is governed by rainfall, rice cropping season and water management. Rice fields should be targeted in the early stages of rice growth (transplanting to early tillering stages) when they are highly productive while majority of the temporary peri-domestic larval habitats should be targeted mostly during the rainy season. Such interventions should consider habitat and site specific attributes of larval productivity. The fact that 'unplanned" rice growing supports more *Anopheles *larvae than planned rice growing system calls for better management of the rice cultivation and subsequent water distribution so as to reduce the active period when the rice fields are flooded.

## Competing interests

The authors declare that they have no competing interests.

## Authors' contributions

JMM, JS EJM and SM conducted all the experimental work. JS provided scientific guidance in data collection, analysis and manuscript preparation and planning, and implementation of day-to-day field activities. WG, BJ, EWK and CMM, offered scientific guidance in data analysis and manuscript preparation. JG and RN provided overall supervision of the study and preparation of manuscript. All authors actively contributed to the interpretation of the findings and development of the final manuscript and approved the final manuscript.
